# Changes in Standing and Walking Performance Under Dual-Task Conditions Across the Lifespan

**DOI:** 10.1007/s40279-015-0369-9

**Published:** 2015-08-08

**Authors:** Jan Ruffieux, Martin Keller, Benedikt Lauber, Wolfgang Taube

**Affiliations:** Department of Medicine, Movement and Sport Sciences, University of Fribourg, Bd de Pérolles 95, 1700 Fribourg, Switzerland; Department of Sport and Sport Science, University of Freiburg, Schwarzwaldstr. 175, 79117 Freiburg, Germany

## Abstract

Simultaneous performance of a postural and a concurrent task is rather unproblematic as long as the postural task is executed in an automatic way. However, in situations where postural control requires more central processing, cognitive resources may be exceeded by the addition of an attentionally demanding task. This may lead to interference between the two tasks, manifested in a decreased performance in one or both tasks (dual-task costs). Owing to changes in attentional demands of postural tasks as well as processing capacities across the lifespan, it might be assumed that dual-task costs are particularly pronounced in children and older adults probably leading to a U-shaped pattern for dual-task costs as a function of age. However, these changes in the ability of dual-tasking posture from childhood to old age have not yet been systematically reviewed. Therefore, Web of Science and PubMed databases were searched for studies comparing dual-task performance with one task being standing or walking in healthy groups of young adults and either children or older adults. Seventy-nine studies met inclusion criteria. For older adults, the expected increase in dual-task costs could be confirmed. In contrast, in children there was only feeble evidence for a trend towards enlarged dual-task costs. More good-quality studies comparing dual-task ability in children, young, and, ideally, also older adults within the same paradigm are needed to draw unambiguous conclusions about lifespan development of dual-task performance in postural tasks. There is evidence that, in older adults, dual-task performance can be improved by training. For the other age groups, these effects have yet to be investigated.

## Key Points

**Table:** 

Older adults show age-related decreases in the performance of postural tasks under dual-task conditions.
The limited literature available suggests a trend towards larger dual-task costs (i.e., decreased performance in one or both tasks) in children compared with young adults.
More studies comparing several age groups within the same paradigm are needed to obtain a conclusive picture of the development of postural dual-task ability across the lifespan.

## Introduction

Everyday life involves numerous situations in which a postural task is performed concurrently with a second task such as walking and carrying a tray with glasses or while talking on the phone. In general, these so-called dual-task (DT) situations are considered rather unproblematic, i.e., they do not constitute a risk for falling, provided the postural task is executed in an automatic way and thus requires little cognitive resources. However, in situations where more attentional resources are needed or where attentional capacities are limited, even seemingly simple motor skills may become problematic when performed simultaneously with an attentionally demanding task. The addition of a concurrent task increases the overall attentional demands and may lead to interference between the two tasks if processing capacities are exceeded, manifested in a decreased performance in one or both tasks. In the case of postural tasks or during walking this may, at worst, result in a fall. Children and older adults are particularly exposed to such risks as postural tasks have been shown to consume more attentional resources in these age groups while these resources are limited compared with young adults [1]. For older adults, age-related differences in DT performance of postural tasks have been shown repeatedly (see Boisgontier et al. [2] for review) whereas much less research has been carried out on this topic in children. However, children and older adults show many parallels in the control of postural tasks.

Postural instability and the incident rate of falls are increased in both children and older adults compared with young adults [3]. It is assumed that this relies at least in part on differential postural control strategies and altered weighting of sensory input [4–6]. Furthermore, the level of automaticity when processing posture-related information is considered to be an important indicator for walking and standing performance and the occurrence of future falls [7, 8].

In general, postural control and walking abilities are considered to be developed during childhood and adolescence, reach their maxima in young adults, and thereafter progressively decline with age (and sedentary behavior). Many systems critical for postural control, including sensory systems (visual, somatosensory, and vestibular), musculoskeletal systems, central processing, and neural pathways are developed during childhood and still maturing during adolescence [6, 9, 10]. However, it is well known that normal aging is accompanied by a decline in the integrity of these same systems [11, 12].

Some aspects of reactive balance control seem to be effective soon after birth. For instance, the magnitudes of head postural responses of 3-day-old infants were shown to be sensitive to optic flow velocity [13]. This strong dependency on visual input for postural control remains during early childhood, nicely demonstrated for example by the moving room experiments initiated by Lee and Aronson [14]. This dependency on visual information and the resultant susceptibility to a manipulation thereof has been explained by the fact that the somatosensory system has not yet been properly ‘calibrated’ in the infant [6]. With increasing age, the somatosensory system becomes more reliable and is gradually integrated in postural control [9]. Nevertheless, it has been shown that adolescents are still less efficient than young adults in integrating available somatosensory information to improve postural control [9, 10]. Interestingly, older adults also exhibit stronger dependency on visual input than young adults when tested in the moving room, possibly owing to an age-related reduction in proprioceptive feedback [15]. Therefore, it is hardly surprising that during walking, DT costs, i.e., the reduction in performance due to the execution of a concurrent task, are especially pronounced in the older adults when concurrent tasks are chosen that require substantial visual processing [16, 17].

Besides the deficits in the sensory systems, the ability to efficiently select and weight sensory inputs seems to be particularly affected by age [9, 11, 18, 19]. Both children and older adults show impaired postural control when the number and/or the quality of sensory inputs is reduced leaving less redundancy of sensory information [6]. It was shown that if the inputs from the visual and the somatosensory systems are experimentally reduced so that the main sensory input is coming from the vestibular system, both young children and older adults have difficulties with balance [6]. Furthermore, compared with young adults, children and older adults show particular deficits in the ability to resolve sensory conflicts [6, 12]. In contexts requiring a fast reweighting of sensory inputs due to sudden changes in the sensory environment, these limitations may increase the risk of losing balance [11, 20], especially in situations where insufficient attentional resources are available for or allocated to the postural task [21].

Another parallel in children and older adults might be the reduced ability to activate muscle synergies. It takes several months to years until children display adult-like muscle synergies while reacting in response to external perturbations [22]. Muscle synergies during anticipatory postural control develop even later: Nashner and colleagues [23] assume that it takes 4–6 years to acquire adult-like anticipatory postural adjustments. Before coordinated muscle synergies are established, increased tonic unspecific co-activation can be observed in children [6, 22]. This is similar to the mechanisms displayed in older adults, in whom loss of synergistic activity is accompanied by increased co-contraction [24]. Concerning muscular activity during gait, several studies indicated intense co-activation of leg muscles, resulting in greater stiffening of leg joints in the older adults (e.g., [25]). Recently, it was assumed that the increase in co-activation in older adults compared with young adults is at least partly due to differences in motor cortical control [26]. With increasing age, the level of cortical reciprocal inhibition is reduced or even absent [27]. This means that in older adults, the afferent input to the agonist muscle does no longer lead to a reduction in corticospinal output of the antagonistic muscle, probably favoring an elevated co-activation. Similarly, the silent period, indicating gamma-aminobutyric acid-B mediated cortical inhibition, was shorter in older adults during challenging coordination tasks [28]. Finally, short-interval intracortical inhibition, representing gamma-aminobutyric acid-A mediated cortical inhibition, was reported to be less pronounced in older adults compared with young subjects [26, 29]. It was highlighted that the decrease in short-interval intracortical inhibition was especially pronounced during challenging postural tasks and was negatively correlated with postural stability [29]. All these studies indicate that aging causes a reorganization of cortical motor control, leading to a decrease in cortical inhibition and a subsequent increase in cortical activation. It is assumed that these changes in motor (cortical) control affect not only the performance of the postural task but also DT performance [2]. Moreover, these age-related changes may often not be detected during single-task performance but appear more clearly when a second task is performed concurrently, requiring additional (cognitive) resources [30].

In children, we are not aware of any studies measuring cortical activation and inhibition during postural tasks or during walking. However, the emerging picture obtained at rest or during non-postural motor tasks is very similar to that of older adults showing reduced inhibitory processes at the cortical level. Garvey and colleagues [31] reported shorter silent periods in children than young adults. With maturation, the silent periods increased and motor task performance, i.e., finger tapping speed, increased. Several studies indicate that short-interval intracortical inhibition is also reduced in children [32, 33]. Interestingly, lower short-interval intracortical inhibition is especially prominent in children with attention-deficit/hyperactivity disorders (ADHD) [34] and children born very preterm [35]. In comparison to healthy peers, children with ADHD and children born preterm demonstrate delayed motor skill development [34, 35].

In summary, there is good evidence that motor strategies for posture and walking change across the lifespan. Furthermore, motor (cortical) control is different in older adults and children compared with young adults and pathological states further increase these differences. In general, inhibitory processes at the cortical level seem to be less pronounced while cortical activity is facilitated. The greater supraspinal activity is often considered as a compensatory strategy to counteract less automatic motor control [2]. However, this allocation of additional resources to ensure postural control and stable walking may have unfavorable consequences as these cognitive resources may no longer be available for other activities. Thus, if the allocation of attentional resources to one particular task A is increased, concurrent performance of a task B is more likely to cause interference. Interference due to less automatic performance of motor skills may therefore be an important contributor to age-related differences in DT performance. However, the total processing resources that are available for task execution also vary across the lifespan and may further aggravate age-related differences in DT performance. In older adults, processing capacities are declined

compared with young adults [36]. Furthermore, when attention needs to be divided, older adults appear less able to allocate available resources in an optimal way [36, 37]. Not only older adults but also children show reduced processing capacities compared with young adults. While in older adults this is due to deteriorations in the functioning of the neural system, in children, these cognitive functions are not yet fully developed [38, 39].

Based on the above-mentioned studies displaying age-specific motor control strategies and differences in the total amount of processing resources, we assumed greater DT costs in older adults and in children compared with young adults.

For older adults, a recent review article indeed points in this direction but emphasizes that these differences are mainly apparent when considering challenging postural tasks [2]. Similarly, DT costs during walking seem to be enhanced in older compared with young adults when the task demands are high; especially when the concurrent task requires considerable visual processing [16, 17, 40]. For children, no (systematic) review articles are available, yet, and the results of different studies are divergent. However, the only study that compared DT costs in children, young and older adults, so far, found a U-shaped relation of DT costs with age [41].

The aim of the present review is to identify age-related changes in standing and walking performance under DT conditions. To this end, we systematically reviewed literature comparing young adults’ performance with that of either older adults or children. A special emphasis was placed on children as this age group has not been systematically reviewed yet. For the analysis, we chose a novel approach that allowed us to include a large number of studies. In an additional section, we elaborate the question if and how DT ability can be trained.

## Methods

### Search Strategy

Electronic bibliographic databases PubMed and Web of Science were searched up to October 2014 for relevant articles. The following combination of search terms was used in both databases: (balance OR postur* OR gait OR walking OR locomotion) AND (dual task*) AND (age OR age* OR aging OR elderly OR old* OR senior OR child* OR adolescent). Additionally, references found in retrieved articles were checked for eligibility. Only English language original articles were considered for this review.

### Study Selection

The objective of this review was to assess the effect of normal development across the lifespan on DT abilities. Thus, all studies comprising patient groups (e.g., Parkinson’s disease, Alzheimer’s disease, dementia, diabetes, ADHD, dyslexia, concussion, vestibular disorder, balance or cognitive impairment) were excluded.

Dual-task studies vary widely with regard to the design (e.g., type and difficulty of the postural/concurrent task, measurement of single-task performance, variables measured, instructions, statistics) making it very difficult and inappropriate to directly compare results of different studies. For instance, DT costs in study A performed in young adults cannot be compared to DT costs of older adults in study B. To be able to draw valid conclusions about age-related differences in DT performance, we therefore only included studies in this review in which at least two different age groups, one being young adults, were evaluated within the same study design.

Studies were included if one task was either a standing or a walking task. The walking task was restricted to ‘normal’ walking. Studies with tasks such as obstacle crossing, stepping tasks, or stair ambulation were excluded as no such studies exist in children. Furthermore, to assess DT effects, only studies evaluating at least the postural task under both single- and dual-task conditions were included in this review. Because of the fact that in many studies comparing children with adults, single-task performance of the concurrent task was regrettably not measured, the absence of this criterion did not lead to exclusion of the study. For the same reason, i.e., to include as many studies with children as possible, no further exclusion criteria, in particular regarding study quality, were applied.

### Data Collection

The above-mentioned large variety of study designs across DT literature leads to considerable differences in the type of results reported. Not only do they derive from various tasks and various parameters measured but they also differ in the way they were calculated and reported. For instance, diverse statistics are used to assess age-related differences in DT performance, such as a significant difference between age groups in the absolute or relative differences between single-task and DT performance, or a significant interaction effect of age group and condition (single-task vs. DT) in an analysis of variance. Effect sizes were often not reported.

Because of these facts and the large number of studies included in this review, the application of a conventional synthesis method or even a meta-analysis was not possible. We therefore chose a different, more global approach for the present review: First, all included studies were scanned for reported significant age-related differences in DT performance. Such age-related differences in DT performance can result from a decreased performance under DT conditions compared with single-task conditions in one group and not in the other or in both groups but significantly more in one group, or, conversely, from an improvement under DT conditions only or significantly larger in one group. Second, numerous studies measured and reported several parameters for one task but often found significant age-related differences in DT performance only for some of them. To allow for this fact, the number of variables for which a significant age-related difference in DT performance was found is always reported relative to the number of variables measured in the respective study. Third, we classified the results into five age ranges. The ranges were defined as follows: (a) young children under the age of 8 years, (b) older children aged between 8 and 13 years, (c) young adults aged between 19 and 35 years that served as the reference population, (d) younger old aged between 60 and 69 years, and finally (e) older old, 70 years and older. The determining factor for the classification was the mean age of the groups. Furthermore, we distinguished between studies that had standing as the postural task and those which had walking as the postural task. Fourth, the distribution of the studies was tested for effects of postural task type or age by means of Pearson’s Chi-square tests. Separate tests were run for both factors (type of task and age) and for age-related differences in DT performance on the postural and the concurrent task. Fifth, owing to the fact that age-related differences in DT performance between children and young adults have not been reviewed in detail yet, a special emphasis was placed on the children part. Specifically, the effect of age (young children vs. older children), the type of tasks, and the difficulty of the tasks were examined in more detail.

## Results

**Fig. 1:**
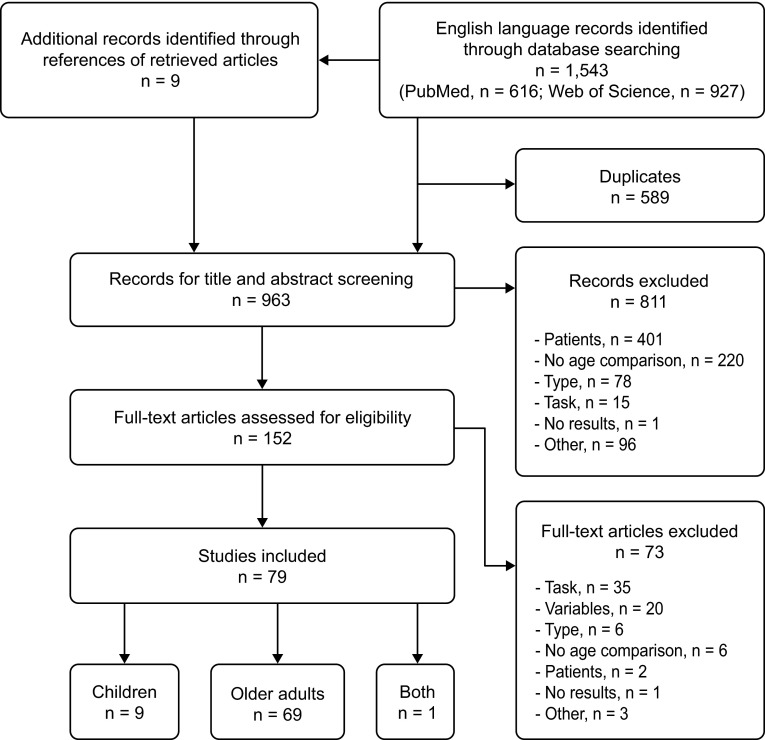
Flow chart of the systematic study selection procedure. The reasons for exclusion were: No age comparison the study did not compare different age groups; No results no results are reported; Other study was not eligible for other reason; Patients the study included patient group(s); Task no standing or walking task; Type publication type was not original article (e.g., review article); Variables no appropriate variables measured or postural task not measured under both single- and dual-task conditions. See text for details on exclusion criteria

**Fig. 2:**
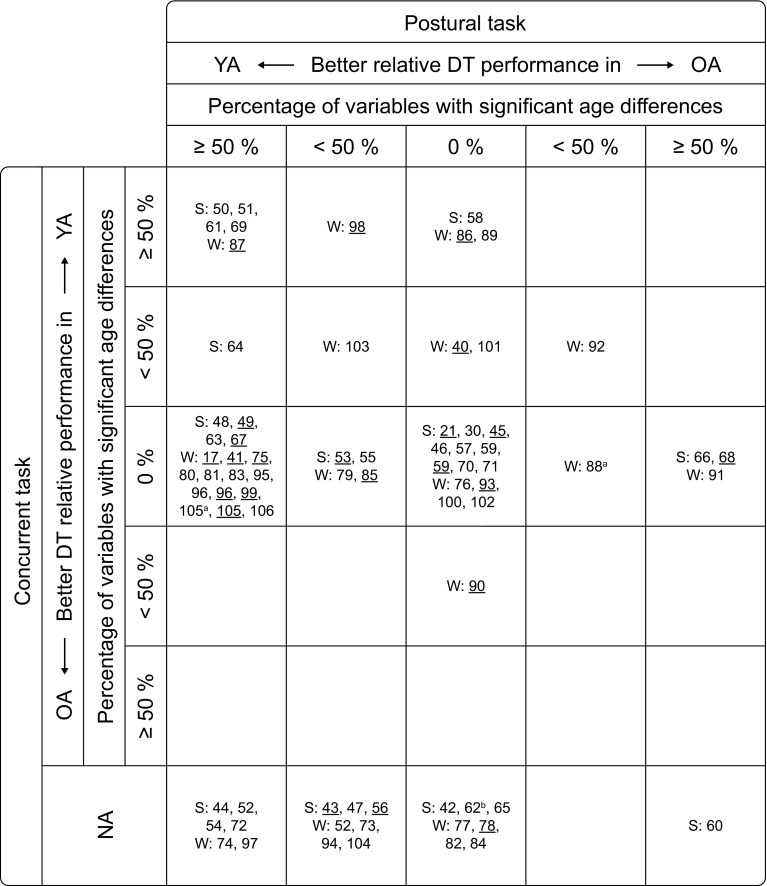
Age-related differences in dual-task (DT) performance (ARD) between young (YA) and older adults (OA). For each study (identified in the figure by its reference number), the number of dependent variables for which significant ARD were found, either in favor of the YA or the OA, were expressed as a percentage of the total number of variables reported, separately for the postural (*columns*) and the concurrent task (*rows*). For each task, the studies were then classified into five ranges: no ARD (0 %), ARD in less than 50 % of the variables, and ARD in 50 % or more of the variables, the latter two with a relatively better DT performance either in YA or in OA. For instance, the *top left* field lists the studies that found a significantly better DT performance in YA compared with OA in at least 50 % of the variables measured, both in the postural and the concurrent task. The studies are further classified by the postural task being standing (S) or walking (W) and the mean age of the older subjects (*underlined* = 60–69 years; not underlined = 70 years or older). *NA* no results for DT performance in concurrent task available. ^a^ data from the same study and same subjects; ^b^ age group not clear (mean age not reported, subjects aged between 65 and 75 years)

**Fig. 3:**
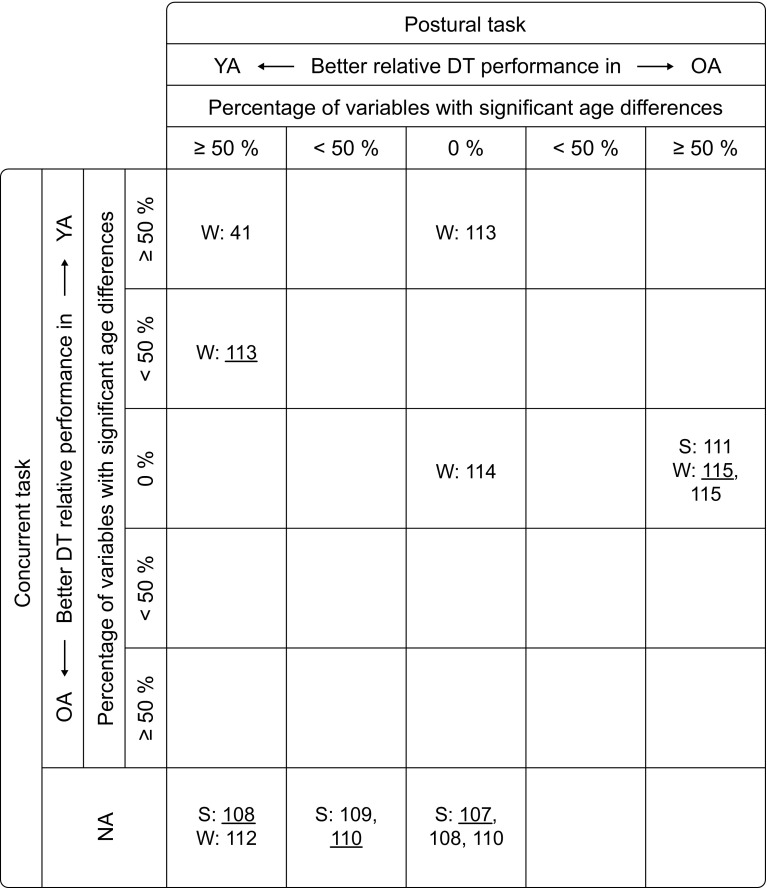
Age-related differences in dual-task (DT) performance (ARD) between young adults (YA) and children (C). For each study (identified in the figure by its reference number), the number of dependent variables for which significant ARD were found, either in favor of the YA or the C, were expressed as a percentage of the total number of variables reported, separately for the postural (*columns*) and the concurrent task (*rows*). For each task, the studies were then classified into five ranges: no ARD (0 %), ARD in less than 50 % of the variables, and ARD in 50 % or more of the variables, the latter two with a relatively better DT performance either in YA or in C. For instance, the *top left* field lists the studies that found a significantly better DT performance in YA compared with C in at least 50 % of the variables measured, both in the postural and the concurrent task. The studies are further classified by the postural task being standing (S) or walking (W) and the mean age of the C groups (*underlined* = young C, <8 years; not underlined = older C, 8–13 years). *NA* no results for DT performance in concurrent task available

The database and reference search identified 963 records for screening. After applying the exclusion criteria, 79 studies were included in this systematic review, of which 70 compared older adults with young adults. Only ten studies were found that compared children with young adults. One study [41] included all three age groups, [41] and therefore appears in both categories. To attempt to increase the very limited number of children studies identified through the first search in October 2014, a second search was conducted only for children studies in January 2015. This second search identified one additional study. The detailed procedure of study selection is depicted in Fig. 1. The studies including older adults are listed in Fig. 2, those including children are listed in Table 1 and Fig. 3.

**Table 1:** Age-related differences in dual-task performance (ARD) between children (C) and young adults (YA) for studies with a standing or walking task as the postural task. Results are reported as the number of dependent variables for which C’s dual-task performance relative to single-task performance was significantly better (C > YA) or worse (C < YA) than that of YA in proportion to the total number of variables measured/reported

Study	Subjects (mean age ± SD/*n*)			Postural task				Concurrent task			Effect of task difficulty
	YC (<8 years)	OC (8–13 years)	YA	Type	TD	Dependent variables	Significant ARD	Type	ST	Significant ARD	
Standing postural tasks											
Olivier et al. [107]	7.3 ± 0.2/8		25.7 ± 2.3/9	Semi-tandem stance without Achilles tendon vibration Semi-tandem stance with Achilles tendon vibration	2 3	ML COP amplitude ML COP velocity	None	Mod. Stroop congruent Mod. Stroop incongruent	–		None
Olivier et al. [108]	7.3 ± 0.2/8	8.2 ± 0.2/8 9.2 ± 0.4/7 10.1 ± 0.1/6 11.4 ± 0.3/8	25.7 ± 2.3/9	Semi-tandem stance without Achilles tendon vibration Semi-tandem stance with Achilles tendon vibration	2 3	ML COP amplitude AP COP amplitude ML COP velocity AP COP velocity	YC < YA in 2/4	Mod. Stroop congruent Mod. Stroop incongruent	–		None
Palluel et al. [109]		12.9 ± 0.7/14	26.1 ± 4.3/13	Semi-tandem stance on firm surface Semi-tandem stance on foam surface	2 3	Ellipse area AP COP velocity ML COP velocity RMS AP COP RMS ML COP	OC < YA in 2/5 during counting	Stroop congruent Stroop incongruent Backward counting	–		ARD for counting, not for Stroop
Reilly et al. [110]	5 ± 1/6	9 ± 2/6	21.5 ± 1.5/6	Wide stance Semi-tandem stance	1 2	ML COP range AP COP range RMS ML COP velocity RMS AP COP velocity	YC < YA in 2/4 for wide stance YC < YA in 1/4 for semitandem stance	Visual working memory	–		Less ARD for semi-tandem than wide stance
Schaefer et al. [111]		9.3 ± 0.3/9 11.5 ± 0.2/9	22.9 ± 1.3/9	Wobble board on stable platform Wobble board on moving platform	4 5	COP area	OC > YA in 1/1	Method of loci n-back (visual)	X	None	None
Walking postural tasks											
Abbruzzese et al. [112]		8.1 ± 1.2/10	26.8 ± 4.9/10	Walking at preferred speed	1	Gait velocity Cadence Step length Base of support % double support Step length variability Step time variability	OC < YA in 2/7 for simple DTs OC < YA in 7/7 for complex DTs	Hold empty pitcher Hold empty tray Hold filled pitcher Hold tray with cup on top	–		More ARD for complex tasks
Boonyong et al. [113]	6.2 ± 0.6/20	10.9 ± 3/20	22.8 ± 2.7/12^a^	Walking at preferred speed Walking with obstacle crossing (obst.)	1 3	AP COM ROM ML COM ROM AP peak linear velocity ML peak linear velocity Gait velocity Stride length Stride time Average step width	YC < YA in 5/8 for both difficulties	Auditory Stroop	X	YC < YA in 1/2 during obst. OC < YA in 1/2 for both tasks	YC < YA for concurrent task only during obst.
Krampe et al. [41]		9.5 ± 0.4/30 11.5 ± 0.3/30	24.3 ± 2.2/30	Walking on narrow track	2	Distance	OC < YA in 0.5/1 (only 9-year-olds)	Semantic fluency	X	OC < YA in 0.5/1 (only 9-year-olds)	–
Schaefer et al. [114]		9.0 ± 0.2/32	25.3 ± 2.9/32	Treadmill walking at preferred speed Treadmill walking at 2.5 km/h	1 1	CV for stride length CV for stride time	None	n-back (1–4, auditory)	X	None	No consistent effects
Schaefer et al. [115]	7.6 ± 0.3/18	9.5 ± 0.3/18	26.6 ± 1.8/18	Treadmill walking at preferred speed Treadmill walking at preferred speed − 30 % Treadmill walking at preferred speed + 30 %	1 1 1	Walking regularity	YC and OC > YA in 1/1 during 2-back	2-back (auditory) 3-back (auditory)	X	None	YC and OC better under 2-back and worse again under 3-back

*AP* anterio-posterior, *COM* center of mass, *COP* center of pressure, *CV* coefficient of variation, *DT* dual-task, *ML* medio-lateral, *Mod.* modified, *OC* older children (age 8–13 years), *RMS* root mean square, *ROM* range of motion, *SD* standard deviation, *ST* single-task measurement, *TD* subjective rating of postural task difficulty (1 = easiest), *X* evaluated, *YA* young adults, *YC* younger children (age <8 years), – not evaluated

^a^Data from a different study

### Absolute vs. Relative Performance

It is important to note that, in absolute terms, both children and older adults generally performed worse than young adults in both the postural and the concurrent task. This is true for virtually all studies, task types, and parameters evaluated. The focus of this review lies on age-related effects on the differences between single-task and DT performances (DT performance relative to single-task performance). Only such effects will be reported and discussed in the following sections.

### Age-Related Differences in Dual-Task Performance Between Older Adults and Young Adults

In Fig. 2, all the studies that compared older adults with young adults are classified by the percentage of dependent variables for which significant age-related differences in DT performance were found for both the postural and the concurrent task. The studies are further classified by the postural task being standing [21, 30, 42–72] or walking [17, 40, 41, 52, 73–106] and the mean age of the subjects (60–69 vs. 70 years or older).

The distribution of the studies was found to be the same for both postural task types and both age groups, *χ*^2^ (4) < 3.2, *p* > 0.05 for all tests. Therefore, subsequent results are averaged across tasks and age groups. Similarly, studies including both younger old and older old found no differences between these two age groups [59, 96, 105]. They are listed separately for younger old and older old in Fig. 2 but will for subsequent analyses be regarded as one cohort. One study [52] included both a standing and a walking task that will be regarded as two different results.

Regarding the postural task, 38 % of the studies reported better relative DT performances in young adults compared with older adults for at least half of the variables, 18 % found better relative performances in some but fewer than half of the variables. In 35 % of the studies, no significant age-related differences in DT performance were found for the postural task, while older adults outperformed young adults in only 9 % of the studies.

With respect to the performance of the concurrent task, the distribution is different. Twenty out of the 69 studies did not report DT effects for the concurrent task, either because concurrent task performance was not measured at all or not measured under single-task conditions. Of the remaining studies, 18 % found age-related differences in DT performance in favor of the young adults in at least half, 10 % in less than half of the variables measured. Contrary to the performance in the postural task, most studies (70 %) found no differences between older and young adults for the relative DT costs in the concurrent task. Only one study (2 %) reported age-related differences in DT performance in favor of older adults for the concurrent task.

This pattern of older adults tending to show more DT costs than young adults for the postural task but similar costs for the concurrent task also becomes apparent when looking at Fig. 2. The largest number of studies appears in the field representing no age-related differences in DT performance for the concurrent task but age-related differences in DT performance in favor of the young adults for the majority of the assessed parameters for the postural task (31 % of the studies reporting results for both tasks). Sixteen percent of the studies found a better DT performance in at least some of the parameters for both postural and concurrent task. Nevertheless, the field with the second largest number of studies (24 %) is the center field, representing studies which found no age-related differences in DT performance for either task. The number of studies that indicated a relatively better DT performance in older adults compared to young adults, on the other hand, is very limited. In total, five studies (10 %) found a better performance in older adults for the postural task while for the concurrent task there is only one study.

### Age-Related Differences in Dual-Task Performance Between Children and Young Adults

A detailed description of the studies comparing DT performance in children and young adults and the corresponding results are provided in Table 1. Additionally, Fig. 3 graphically illustrates the results, in the same way as Fig. 2 demonstrates for older adults. We here differentiated between young children (aged <8 years) and older children (aged 8–13 years).

As opposed to older adults, only very few studies investigated age-related differences in DT performance between children and young adults. Five studies were identified for standing [107–111] and five studies for walking [41, 112–115] as the postural task. Half of these ten studies did not report DT effects on performance of the concurrent task, four of them using standing as the postural task. This means that there is only one study with standing as the postural task that reported results for DT performance in both the postural and the concurrent task. As a result, it was not possible to differentiate studies according to the type of postural task. It should further be mentioned that four of the studies with standing as the postural task had rather small sample sizes (*n* < 10).

Not taking into account differences between young children and older children, six out of ten studies found age-related differences in DT performance in favor of young adults for the postural task, two found no age-related differences in DT performance, and two reported a relatively better performance in children. Regarding DT performance in the concurrent task, no study showed a better performance in children compared to young adults. However, only two studies found a better performance in young adults, both with walking as the postural task. As mentioned before, half of the studies did not provide results on concurrent task performance. In the following sections, these results will be analyzed in more detail with regard to effects of age and task type.

#### Young Children vs. Older Children

The overall picture regarding differences between young children and older children is not clear. Studies with young children tend to be more on the left side in Fig. 3, indicating a worse performance compared to young adults, while studies with older children tend to be more evenly distributed. However, the only study [41] that found a significantly worse DT performance in children compared with young adults in more than half of the variables measured, both in the postural and the concurrent task, examined older children (though the differences were only apparent in 9-year-old children and not in 11-year-old children). Additionally, the study with the oldest children (aged 12–13 years) [109] found that they performed worse under DT condition than young adults. Altogether, there are too few studies with too heterogeneous designs to allow conclusions about differences between young children and older children. We therefore analyzed the four studies that included both age groups [108, 110, 113, 115]. For the postural task, older children outperformed young children in three out of these four studies, one study found no difference [115]. For performance of the concurrent task, one study found no difference between the two groups [115]. The only other study measuring concurrent task performance revealed that young children performed better than older children during an easy postural task (level walking) but no differences were observed for the more difficult postural task (obstacle crossing) [113].

#### Effect of Postural Task Type

For standing as the postural task, it is difficult to draw conclusions about the effect of task type and the task complexity. The only study [111] using a dynamic balance task, namely standing on a wobble board either on stable ground or on a moving platform, found age-related differences in DT performance in favor of the children. All other studies used a static semi-tandem stance (one foot behind the other with the big toe of the rear foot touching the side of the heel of the front foot) as the postural task, which might be considered easier than the dynamic balance tasks mentioned above. They found either no significant age-related differences in DT performance [107] or age-related differences in DT performance in favor of young adults [108–110]. One study additionally tested the subjects in a wide stance task, which is easier to perform than the semi-tandem stance [110]. Interestingly, compared with young adults, young children showed worse performance under DT conditions in this easier standing condition. Application of Achilles tendon vibration [107, 108], standing on a foam instead of a firm surface [109], and standing on a wobble board that is on a moving rather than a stable platform [111] had no effect on age-related differences in DT performance.

Of the studies using a postural walking task, two used a task that differed from normal walking. The first consisted of walking while crossing an obstacle [113], the second consisted of walking on a narrow track [41]. The former found a small effect of task difficulty in that young children and not young adults showed DT costs for the concurrent task but only for the more difficult obstacle crossing task and not for normal walking. For older children as well as for performance of the postural task, no effects owing to task difficulty were found. In the latter study, 9-year-old children performed worse than young adults under DT conditions in both postural and concurrent task, 11-year-old children did not. Of the three studies using ‘normal walking’ tasks, one showed no age-related differences in DT performance [114], one showed age-related differences in DT performance in favor of young adults [112], and one showed age-related differences in DT performance in favor of young children and older children with a simple DT [115].

#### Effect of Concurrent Task Type

Five studies used a concurrent task that involved the visual system (three used a Stroop test [107–109], one used a visual working memory task [110], and one used a visually presented n-back task [111]), all of them concurrently with a standing task. In two of them [107, 110], young children performed worse than young adults in the postural task (DT effects were not measured for the concurrent task), in one [111] older children outperformed young adults only in the postural task, the other two studies showed no age-related differences in DT performance. Two other, non-visual, concurrent tasks were used in combination with standing as the postural task. Using a backward counting task, one study [109] found a decreased performance in older children compared with young adults in the postural task (DT effects not measured for concurrent task). In the same study, no age-related differences in DT performance were found with a Stroop task. With an episodic memory task using the method of loci, Schaefer and colleagues [111] found age-related differences in DT performance in favor of older children compared with young adults for performance of the postural task only. No difference between this episodic memory task and a visual n-back task was found.

In two further studies, an auditory n-back task was used concurrently with walking as the postural task. While one [114] found no age-related differences in DT performance for either task, the same group found, in another study [115], an increase in walking regularity from single-task walking to walking while performing a 2-back task in young children and older children but not in young adults. However, when performing a 3-back task, both young children and older children became more irregular again. No effects were found for young adults or for performance in the concurrent task in this study. Only one study investigated age-related differences in DT performance between children and young adults using motor concurrent tasks [112]. Two simple and two complex tasks were used during walking at a preferred speed. The simple tasks consisted of holding an empty pitcher and carrying an empty tray. The complex tasks were holding a filled pitcher and carrying a tray with a cup on top. The authors found more age-related differences in DT performance for walking in favor of the young adults for the complex DTs (7/7 variables) than for the simple DTs (2/7). Performance of the concurrent tasks was not measured.

## Discussion

The aim of the present review was to assess differences in the performance of standing and walking tasks under DT conditions across the lifespan. Studies comparing young adults’ DT performance to that of children and older adults were systematically reviewed.

### Young Adults vs. Older Adults

The results of the studies comparing DT performance of young adults and older adults show that older adults tend to perform worse under DT conditions than young adults. More than half of the studies included in this review found age-related differences in DT performance in favor of the young adults for performance of the postural task, for the concurrent task, 28 % did so. Very few studies found older adults to perform better than young adults. However, more than a third of the studies found no age-related differences in DT performance between older and young adults for postural task performance and 70 % for concurrent task performance. These findings are in line with those of a recent well-conducted review article on differences in performance of postural DTs between young and older adults [2]. Boisgontier and colleagues differentiated between postural tasks in stable and unstable conditions and found significant differences in DT costs between young and older adults, with very few exceptions, only in unstable conditions, although there often was a trend towards higher costs in older adults also in stable conditions. In that review, the authors only included studies that reported postural and concurrent task performance in both single- and dual-task conditions and concentrated exclusively on balance tasks. The present review, having less tight inclusion criteria and including not only standing but also walking tasks, offers a less detailed but in return more complete overview of studies investigating age-related differences in DT performance of postural tasks. The results confirm the general picture that older adults show age-related deficits in DT performance compared with young adults [2, 116]. Confirmation of previous findings is, in fact, valuable as results of systematic reviews in the field of DT studies are not always consistent. Different inclusion criteria (e.g., population, tasks, and parameters) often lead to divergent and even contrary results. Thus, the present review being in line with previous results, despite the different approach used, is good evidence for age-related differences in DT performance between healthy young adults and older adults.

One possible explanation for the fact that still an important number of studies found no age-related differences in DT performance between young and older adults is that the differences and/or the number of participants were too small to reach significance. This explanation is supported by the above-mentioned review [2] and is especially evident for simpler tasks. Thus, the different task difficulties used in the studies are another possible explanation. It was shown that age-related differences are more pronounced in more complex DT situations [2]. It is conceivable that age-related differences in DT performance in simple DTs are too small to be detected. Alternatively, they may not even be present at all because overall attentional demands might not exceed processing capacities and therefore cause no interference. Although task difficulty is certainly an important point affecting DT performance, the effect of task difficulty on age-related differences in DT performance between young and older adults was not evaluated in this review owing to the large number of incorporated studies.

The general picture (Fig. 2) suggests that, relative to young adults, older adults tend to prioritize the concurrent task under DT conditions. The number of studies that found age-related differences in DT performance in favor of the young adults is larger for the postural than for the concurrent task. Furthermore, the field in Fig. 2 with the largest number of studies is the one representing studies that reported no age-related differences in DT performance for the concurrent task but significant age-related differences in DT performance in favor of the young adults in more than half of the parameters for the postural task. Shumway-Cook and colleagues [65] hypothesized that posture would be given a priority when performed concurrently with a cognitive task, the so-called posture first model. However, contrary to their hypothesis, they found in their study that subjects prioritized the concurrent task. As a consequence the authors suggested a modification to the model to acknowledge the complexity of task prioritization. They suggested that task prioritization was dependent on multiple factors such as the type of the tasks, the goal, and the instructions and that a pure posture first strategy is only adopted in situations where postural stability is at risk. A newer model proposed by Yogev-Seligmann and colleagues [117] follows the same lines but focuses more on intrinsic factors such as one’s assessment of the individual capabilities (postural reserve) and the postural threat (hazard estimation). According to these models, the current results suggest that the postural tasks used in the studies were no threat to participant’s stability or not perceived as such. At least, the overall results show no evidence for a posture first strategy in older adults. There are studies showing even more DT costs for the postural task in older adults when the postural task becomes more challenging while costs for the concurrent task remain stable [48, 67]. This suggests that even more challenging tasks, if not perceived as a threat, do not lead to more attention being allocated to the postural task. However, when interpreting the results reported in the present review, one has to keep in mind that they are relative differences in DT costs between young adults and older adults rather than absolute DT costs.

An important point regarding age-related differences in DT performance is the fact that under single-task conditions, older adults generally perform worse than young adults in postural tasks. In other words, they perform closer to their individual postural stability boundaries. As a consequence, the same amount of interference, caused by the concurrent performance of a concurrent task, can lead to a highly increased risk of falling in older adults while it poses no threat to postural stability of young adults. This should not be neglected when talking about implications of DT interference on everyday life.

### Young Adults vs. Children

Compared with older adults, only very few studies exist that investigated age-related differences in DT performance between children and young adults. Moreover, half of them (*n* = 5) had samples with no more than ten participants per group. In addition, also half of the studies did not assess single-task performance of the concurrent task, which makes it impossible to calculate DT effects for this task. Thus, in these studies, DT effects in the postural task can only be interpreted with the reservation of possible unknown changes in concurrent task performance. For both of these limitations, i.e., small sample sizes and no assessment of single-task performance of the concurrent task, four of the five concerned studies used standing as the postural task. This makes it difficult to draw valid conclusions for this type of task.

The distribution pattern of the studies comparing children’s and young adults’ DT performance (Fig. 3) is not clear-cut. If all studies are considered, the pattern roughly resembles the one found for the comparison of young adults and older adults (Fig. 2). However, if only studies are considered that report DT costs for both the postural and the concurrent task, there is no evidence for consistent age-related differences in DT performance between children and young adults. Not taking into account differences between young children and older children, six studies found children to perform worse under DT conditions compared with young adults for postural task performance (four of which did not report DT costs for the concurrent task), while two studies found no age-related differences in DT performance (one reporting DT costs for the concurrent task). Interestingly, two studies reported a relatively better DT performance in children compared with young adults. For performance of the concurrent task, two of five studies reporting DT effects found age-related differences in DT performance in favor of the young adults, three found no age-related differences in DT performance, while there is no study showing a better performance in children compared with young adults for the concurrent task.

Results show a tendency for improvements with age in children, at least for postural task performance. However, no statement can be made as to the age at which children’s performance reaches the level of young adults. There is, on the one hand, a study showing that children aged 12–13 years still perform worse than young adults under DT conditions [109] and, on the other hand, a study in which children aged 7 years outperformed young adults, at least under the simpler DT conditions [115].

As for effects of type or difficulty of the tasks, no statement can be made. However, there was no evidence for a systematic influence of task type or difficulty on age-related differences in DT performance. Contrary to what might be expected due to children’s strong dependence on visual input for postural control, there also is no evidence that children necessarily show larger performance decrements with DTs that involve the visual system. However, we need to note here that in all these studies the visual concurrent task was combined with a standing postural task. Dependence on the visual system and thus the influence of a visual concurrent task might be larger in walking [40].

In summary, we can say that there is feeble evidence for age-related differences in DT performance between children and young adults in favor of the latter but only for the postural task and only in some situations. Furthermore, DT performance seems to be improved with age in children. To obtain a clearer picture of age-related differences in DT performance between children and young adults, further high-quality studies are needed.

### Dual-Task Performance Across the Lifespan

On the basis of age-related changes in motor control strategies and cognitive resources we hypothesized to find a U-shaped relation for DT costs as a function of age, with higher costs in children and in older adults. For older adults, our hypothesis was fully confirmed showing increased DT costs in this age group compared with young adults. For children, the influence of dual tasking has to be clarified by further studies. So far, a slight trend towards enlarged DT costs in children compared with young adults can be assumed.

For older adults, it seems that processing of posture becomes more cognitively controlled with aging and thus recruits more attentional resources [2]. In the case of the postural task being challenging and/or being combined with an attentionally demanding concurrent task, the required attentional resources may exceed older adults’ (limited) resources. Furthermore, even seemingly simple tasks may be executed in a less automatic manner and therefore demand more cortical involvement, further limiting the available cognitive resources [2].

For children, similar mechanisms may be at work, i.e., lower cognitive resources and less automatic skill execution, but they are less well investigated. In contrast to older adults, in whom deterioration of formerly efficient structures takes place, the reason for age-related differences in DT performance in children are related to a not yet fully developed (neural) system.

### How to Improve Dual-Task Performance in Different Age Groups?

So far, this systematic review illustrated the development of standing and walking performance under DT conditions across the lifespan. In this section, the influence of training interventions on DT performance will be briefly (non-systematically) outlined. Regrettably, owing to the lack of studies investigating the effect of training on DT performance in young adults and children, no comparisons between different age groups could be made. This part will therefore focus on training effects in older adults.

First of all, it is important to note that there is a general consensus that DT performance can be improved by training (as reviewed by several authors [118–123]). However, results of existing review articles are not consistent and depend on the inclusion criteria regarding for instance the study populations (healthy vs. impaired) and the type of the intervention (single-task vs. DT training). When only considering reviews that exclusively included healthy older adults and that differentiated between the effects of single-task and DT exercises, two systematic reviews [118, 122] showed differences in the effectiveness of single-task and DT interventions on DT performance. The first review came to the conclusion that single-task training does not transfer to DT performance [118]. Similarly, the second review by Wollesen and Voelcker-Rehage [122] proposed that the best training effects on both cognitive and motor performance under DT conditions can be expected when performing a DT training, provided that these DT interventions “include a certain level of exercise load such as arising difficulties, a certain duration and level of task specificity to gain task related adaptations, and variable task prioritization of the training tasks”. However, when taking into account not only healthy seniors but also people with neurological impairments, Pichierri and co-workers [119] could not identify advantages of a DT training over a single-task training. Thus, health status may greatly influence the responsiveness towards a training program with DTs.

Two recent well-conducted studies [124, 125] in older adults support and extend the findings of these systematic reviews. The authors showed that training under single-task as well as under DT conditions can be equally effective at improving balance performance in single-task contexts. When a DT was added, however, only participants who participated in the DT training showed improved performance. As the ability to purposefully direct attention may play an important role in the acquisition of DT coordination skills [126], the authors compared two different types of DT interventions. The variable-priority group was required to vary their priorities between the two tasks (postural and cognitive tasks), whereas the fixed-priority group was asked to equally emphasize both tasks. Only the variable-priority group showed beneficial training adaptations in gait speed under DT conditions after 2 weeks and maintained these effects at a 12-week follow-up [124]. Thus, not only single-task vs. DT training may influence the training outcome but also how subjects are instructed to direct their attention during the execution of DTs. It is also worth mentioning that improved DT skills are specific and not necessarily transferable to a novel DT situation [125].

These studies, solely conducted in older adults, show that depending on the training regime and the instruction, DT performance is differently affected. The next important step would be to compare these effects with training effects in young adults or children. However, this is not possible because of the lack of studies in the latter age groups. We included this section to highlight this lack of knowledge.

### Limitations

The present review is not without limitations. In an attempt to give as complete an overview as possible, we included a large number of studies. Because of this large number of included studies, specifically with older adults, the approach we chose is more global. Particularly, no detailed analysis was performed in older adults with regard to the effect of different task types and difficulties. In addition, the results are reported from a more global perspective. We did not evaluate absolute DT effects nor did we consider whether they were costs (in terms of a decreased performance under a DT condition compared with a single-task condition) or an improvement in performance. We only report age-related differences in these DT effects. Owing to the fact that we classified the studies by the percentage of the measured parameters for which significant age-related differences in DT performance were found, the classification of a study largely depends on the number of parameters measured (reported). However, classification of the study also clearly depends on the type of parameters measured. This can nicely be shown using the example of two papers reporting different results of the same well-conducted study [88, 105]. The task consisted in walking on a treadmill while performing n-back tasks. The first paper about this study [88] reported results of spatiotemporal gait parameters (stride time and length, step width, and velocity) and variability thereof. They found age-related differences in DT performance for two parameters: older adults reduced their variability of stride-length and velocity from single-task walking to walking while performing a 1-back task while in young adults this was not the case. When working-memory load was further increased (2-, 3-, 4-back), young adults rather reduced variability in two parameters. This was not the case for older adults but they still performed better under the 4-back condition than under the single-task walking condition. Summing up, older adults reduced variability under DT conditions more than young adults in two out of eight parameters, at least for the easiest cognitive task. The second paper of the same study [105] investigated regularity of whole-body movements using principal component analysis; young adults and older adults showed similar improvements in regularity from single-task walking to walking while performing a 1-back task. However, young adults further improved regularity with increasing difficulty of the cognitive task, while older adults’ regularity returned to single-task level. Both studies found no age-related differences in DT performance of the concurrent task.

This dependence on the choice of the parameters measured is a basic problem when it comes to interpreting results of postural measures. There are a number of further methodological issues that need to be considered when interpreting results of postural DT studies. They have been discussed in a well-conducted review article by Fraizer and Mitra [127]. In particular, they place emphasis on the aforementioned fact that there is still no consensus as to which parameter best describes postural stability. Furthermore, changes in standing or gait parameters under DT conditions do not necessarily signify a decreased performance in terms of a more unstable posture. Thus, the above-mentioned authors concluded that “changes in postural sway may reflect things other than changes in stability, especially under unperturbed conditions when the system [is] far from stability boundaries”. For instance, postural task execution can, in some cases, also facilitate performance of the concurrent task [127].

Another important issue that is often disregarded is the way in which baseline, or single-task, performance of the postural task is measured. Often this is done in a ‘pure’ single-task condition with no cognitive task. This can be dangerous as in this case we have no control over the cognitive load because we do not know what the participant is thinking about. Changes in performance after addition of an explicit concurrent task could be the result of a change in the type of cognitive load rather than a simple addition of cognitive load [127]. Moreover, changes in postural performance could be due to a change in the focus of attention. Focusing on a highly automated task such as standing or walking, as it is often the case in single-task conditions, is rather unnatural. Directing participant’s attention toward such a task may lead to a shift in the focus of attention from an external to an internal focus. This in turn can lead to changes in the postural control strategy and consequently to changes in performance [128]. The addition of a concurrent task to such a ‘pure’ single-task could draw participant’s attention away from the postural task and thus lead to a more automatic (and possibly more natural) postural control strategy. This can result in changes in postural performance that are not related to the increased cognitive load per se but rather to a shift in the attentional focus. It could be speculated that a simple postural task requires even less cognitive resources under DT conditions because it is performed more automatically. A good approach to overcome these problems would be to control the cognitive load by asking participants to perform a simple attentionally non-demanding task concurrently with the postural task. Thus, a condition with very low cognitive load would be compared with one or more conditions with higher cognitive loads, presumably with a similar focus of attention.

A last issue that shall be discussed here is the influence of different single-task performance levels on age-related differences in DT performance. The problem here is threefold: First, different single-task levels lead to different DT costs depending on whether they are calculated as absolute or percentage costs. Second, if a task is too easy or too difficult for one age group, ceiling or floor effects may come into play and bias results. Third, depending on the individual cognitive (and motor) capacities, one and the same task represents a different attentional load to each participant, especially in different age groups. To prevent the three above-mentioned issues, tasks should be titrated for individual performance levels. Thus, a comparable cognitive load and performance level can be achieved and DT costs can be attributed to the ability of performing two tasks concurrently rather than to differences in single-task performance of the component tasks [129]. It has been suggested that reported age-related differences in DT performance in children and older adults may often be due to the single-task difficulty not being adjusted to individual ability levels [129].

All these above-mentioned limitations should also be considered when conducting studies with the aim to improve DT performance by training. Furthermore, when considering training studies to improve DT performance, it becomes evident that the outcome of systematic reviews largely depends on the inclusion criteria. Thus, it seems important that systematic reviews with different criteria come to the same conclusion before general deductions are made.

## Conclusion

The present systematic review adds to the evidence that older adults show age-related decreases in the performance of postural tasks under DT conditions. Processing of posture seems to become more cognitively controlled with aging and thus require more of the limited attentional resources. In children, the limited literature available suggests a slight trend towards enlarged DT costs in children compared with young adults, which seem to be improved with age. Similar to older adults, lower cognitive resources and less automatic skill execution may be responsible for this difference. While in older adults these effects are due to the degradation of existing structures, these neural structures are still under development in children. Further studies are strongly needed to clarify the influence of dual tasking in children and to shed light on the underlying mechanisms. To get a conclusive picture of the development of DT ability across the lifespan, more studies comparing several age groups within the same paradigm are needed. Ideally, such studies additionally vary factors such as the type of the tasks, task difficulty, or instruction on prioritization within the same study and populations to gain a more detailed insight into the effect of such factors on age-related differences in DT performance. There are a number of methodological issues that need to be considered when designing DT studies in different age groups: First, differences in single-task performance levels should be accounted for. Second, the cognitive load during baseline measurement of the postural task should be controlled and the difficulty of the tasks should be adapted to the participant’s differing cognitive (and motor) capacities to obtain a comparable cognitive load in all participants. Third, both postural and concurrent tasks should always be measured under both single-task and DT conditions. Thus, DT costs can be calculated for both tasks and differences in prioritization can be detected. Finally, results depend on the parameters measured and there is still no consensus as to which parameter best describes postural stability. Such factors could be responsible for the discrepancies that are found between previously reported DT results. These issues have substantially been described in the ecological approach to studying DT performance in different age groups proposed by Li and colleagues [130] and well summarized in a review by Schaefer [1].

In healthy older adults, DT performance can be improved by training. Evidence suggests that DT training leads to better improvements than single-task training and that improved DT skills do not necessarily transfer to novel DT situations. Effects of training on DT performance in young adults and children and potential age-related differences have yet to be investigated.
